# Biotherapeutic Antibodies for the Treatment of Head and Neck Cancer: Current Approaches and Future Considerations of Photothermal Therapies

**DOI:** 10.3389/fonc.2020.559596

**Published:** 2020-11-26

**Authors:** Mohammed M. Al Qaraghuli

**Affiliations:** ^1^ SiMologics Ltd., Glasgow, United Kingdom; ^2^ Department of Chemical and Process Engineering, University of Strathclyde, Glasgow, United Kingdom

**Keywords:** antibody, head and neck cancer, photothermal therapy, gold nanoparticles, gold nanorods

## Abstract

Head and neck cancer (HNC) is a heterogeneous disease that includes a variety of tumors originating in the hypopharynx, oropharynx, lip, oral cavity, nasopharynx, or larynx. HNC is the sixth most common malignancy worldwide and affects thousands of people in terms of incidence and mortality. Various factors can trigger the development of the disease such as smoking, alcohol consumption, and repetitive viral infections. HNC is currently treated by single or multimodality approaches, which are based on surgery, radiotherapy, chemotherapy, and biotherapeutic antibodies. The latter approach will be the focus of this article. There are currently three approved antibodies against HNCs (cetuximab, nivolumab, and pembrolizumab), and 48 antibodies under development. The majority of these antibodies are of humanized (23 antibodies) or human (19 antibodies) origins, and subclass IgG_1_ represents a total of 32 antibodies. In addition, three antibody drug conjugates (ADCs: telisotuzumab-vedotin, indatuximab-ravtansine, and W0101) and two bispecific antibodies (GBR 1372 and ABL001) have been under development. Despite the remarkable success of antibodies in treating different tumors, success was limited in HNCs. This limitation is attributed to efficacy, resistance, and the appearance of various side effects. However, the efficacy of these antibodies could be enhanced through conjugation to gold nanoparticles (GNPs). These conjugates combine the high specificity of antibodies with unique spectral properties of GNPs to generate a treatment approach known as photothermal therapy. This approach can provide promising outcomes due to the ability of GNPs to convert light into heat, which can specifically destroy cancer cells and treat HNC in an effective manner.

## Incidence, Etiology, and Treatment

Head and neck cancer (HNC) affects over 830,000 patients worldwide, and about 430,000 people had died from this disease in 2018 (1). This disease is highly heterogeneous and can affect the hypopharynx, oropharynx, lip, oral cavity, nasopharynx, or larynx. It is the sixth most common cancer, and associated with a high recurrence and poor 5-year survival rate (40–50%) (2). Tobacco and alcohol use are the main risk factors that increase the incidence of HNC (3). Strains 16 and 18 of human papillomavirus (HPV) are also linked with several cases of HNC (4), however, HPV^+^ cancers have a better survival rate and reduced risk of recurrence compared with HPV^−^ (4).

The most common treatment modalities for HNCs include surgery, radiotherapy (RT), chemotherapy (CT), and biotherapeutic antibodies. Early-stage tumors can be treated with single modality treatment such as surgery or RT (5), for patients with advanced-stage disease are normally treated with combined-modality therapy (6). Both CT and RT can be efficacious in treating HNCs, however, patients must withstand their severe side effects (7, 8). Surgery involves resection of the primary tumor with or without lymph nodal dissection, which can have a significant impact on eating, drinking, and talking, and patients will also need to cope with subsequent facial disfigurement (9). In certain cases surgery may involve removing the larynx, which will greatly affect communication (10), and have a negative impact on the patient’s psychology and quality of life. The Food and Drug Administration (FDA) has approved different CTs, such as cisplatin, carboplatin, 5-fluorouracil, docetaxel, methotrexate, and bleomycin, and three monoclonal antibodies (cetuximab, nivolumab, and pembrolizumab) for the treatment of HNCs. The current treatment standard for recurring or metastatic HNCs is based on cetuximab and platinum based cisplatin or carboplatin CT plus methotrexate and 5-flurouracil; which is further strengthened by surgery and RT, and occasionally augmented by paclitaxel and docetaxel (11). Cisplatin resistant, recurring, or metastatic HNCs could be treated through the inclusion of checkpoint inhibitor antibodies, pembrolizumab or nivolumab (12, 13).

Various review articles have focused on different treatment options for HNCs such as surgery (14–16), RT (17–20), CT (21–24), and immunotherapies (25–27). This article will specifically focus on analyzing biotherapeutic antibodies that are currently approved or being examined in different clinical trials. The main targets that have attracted several developed antibodies were analyzed in term of structural illustration and mechanism of action. In addition, the future perspective of using nanotechnology to enhance the efficacy of these antibodies is briefly outlined.

## Antibody Structure

**Figure 1:**
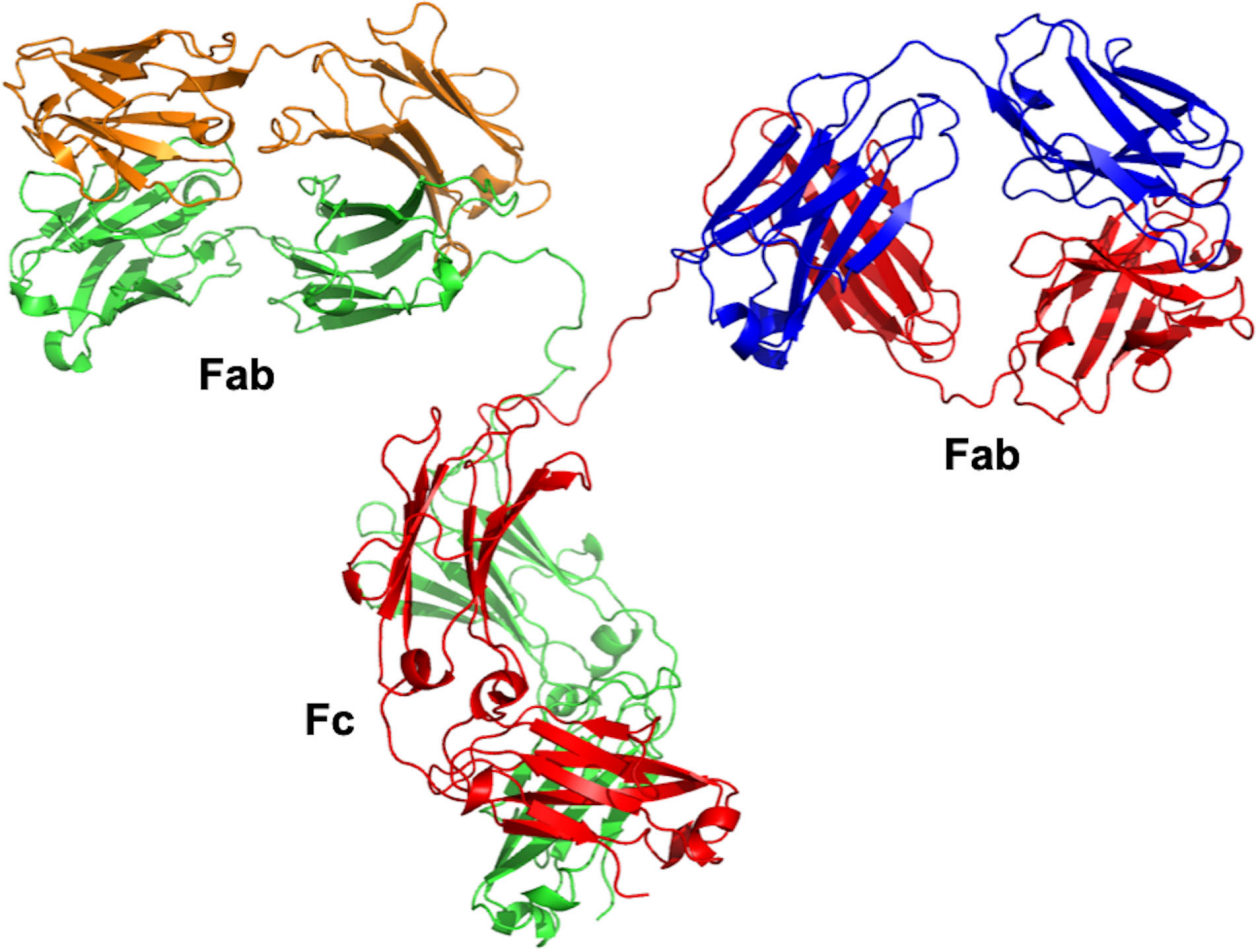
Overall structure of IgG antibody. The crystal structure was obtained from the Protein Data Bank (PDB entry 1IGT). The IgG antibody is composed of two heavy chains (red and green) and two light chains (orange and blue). The crystal structure was viewed and analyzed using PyMOL (The PyMOL Molecular Graphics System, Version 2.4.0 Schrödinger, LLC.).

B-lymphocyte cells are instructed by numerous immunogens, such as bacteria, viruses, fungi, parasites, cellular antigens, chemicals, and synthetic substances to differentiate into plasma cells (28). These plasma cells secrete glycoproteins, also known as antibodies, to protect our bodies against these antigens. The “Y” shaped antibody is generally comprising two heavy and two light polypeptide chains linked together by disulfide bonds. The light chain, which could be lambda (λ) or kappa (k), can be linked to any of the nine characterized heavy-chain subtypes to generate one of the antibody subclasses in humans (IgG_1–4_, IgA_1–2_, IgM, IgE, IgD) (29). The IgG class represents the majority of the licensed therapeutic antibodies and those still in the stage of development (30). The IgG antibody is composed of two identical antigen-binding fragments (Fabs) and one crystallizable region fragment (Fc) (**Figure 1**). Each Fab contains the first two domains of the heavy (VH and CH1) and light (VL and CL) chains, while the Fc region consist of two N-glycosylated CH2 and two CH3 domains (31). Antibodies are well known for their high specificity and selectivity that make them indispensable medicines to treat various diseases, especially cancer, and currently represent a major component of the pharmaceutical industry (32).

## Antibodies Against Head and Neck Cancer

Over the last two decades, our understanding of the molecular mechanisms underlying HNCs, and developments in molecular biology, have led to the development of different targeted therapeutic agents. HNCs can be generally prompted by a primary lesion or metastasize from another cancerous site leading to malignant tumor. Cancer stem cells are constantly addressed as one of the primary mediators of tumor aggressiveness, relapse, and malignancy (33, 34). Tumor-targeting antibodies are generally used to recognize tumor-specific membrane proteins, and induce tumor-killing through Fc-driven innate immune responses, or block cell signaling (35).

Currently and to the best of our knowledge, there are 51 antibodies are being tested at preclinical or clinical trials against HNCs (**Table 1**). Of three of the tested antibodies (nivolumab, cetuximab, and pembrolizumab) were approved by regulatory authorities for the treatment of HNC. The main addressed targets were epidermal growth factor receptor (EGFR) (eight antibodies), programmed cell death protein 1 (PD-1) (six antibodies), programmed death-ligand 1 (PD-L1) (five antibodies), and receptor tyrosine-protein kinase erbB-3 (HER3) (four antibodies). In addition, two antibodies are being developed to each of the cytotoxic T-lymphocyte-associated protein 4 (CTLA-4), HER2, c-Met, and vascular endothelial growth factor A (VEGF-A). Moreover, the developed antibodies were mainly of sub-class IgG_1_ (32 antibodies), followed by subclasses IgG_4_ (11 antibodies) and IgG_2_ (4 antibodies). The antibodies were developed as chimeric, humanized, human, or fusion proteins, which were represented by 5, 23, 19, and 2 antibodies, respectively. Besides the monoclonal antibodies, three antibody drug conjugates (ADCs: telisotuzumab-vedotin, indatuximab-ravtansine, and W0101) and two bispecific antibodies (GBR 1372 and ABL001) are also under development. The analysis will focus on two main targets that attracted the majority of the developed antibodies, as will be detailed in the following two sections.

**Table 1:** List of approved and underdevelopment antibodies against head and neck cancer (HNC).

	Product name	Involved companies	Class	Target	Type	Development stage (ClinicalTrials.gov Identifier, active and recruiting)
1	Cetuximab	MedImmune (AstraZeneca), Merck & Co Inc, Bristol-Myers Squibb AB	IgG1	Epidermal growth factor receptor (EGFR)	Chimeric	In 2006, the FDA has approved cetuximab in 2006 and currently is being used for: •Locally or regionally advanced squamous cell carcinoma of the head and neck in combination with radiation therapy. • Recurrent locoregional disease or metastatic squamous cell carcinoma of the head and neck in combination with platinum-based therapy with fluorouracil. • Recurrent or metastatic squamous cell carcinoma of the head and neck progressing after platinum-based therapy.
2	Pembrolizumab	Merck & Co Inc	IgG4	Programmed cell death protein 1 (PD-1)	Humanized	The FDA approved pembrolizumab in 2019 as a first-line treatment of patients with metastatic or unresectable recurrent head and neck squamous cell carcinoma.
3	Nivolumab	Bristol-Myers Squibb (Medarex) and Ono Pharmaceutical	IgG4	PD-1	Human	The FDA approved nivolumab in 2016 for patients with metastatic or recurrent squamous cell carcinoma of the head and neck (SCCHN) following progression on platinum-based therapy.
4	Toripalimab	Shanghai Junshi Biosciences Co., Ltd	IgG4	PD-1	Humanized	NCT04126460 (phase II) NCT04164238 (phase II) NCT03952065 (phase III) NCT02915432 (phase I/II)
5	Bevacizumab	Genentech (Roche)	IgG1	Vascular endothelial growth factor A (VEGF-A)	Humanized	NCT01588431 (phase II) NCT00588770 (phase III) NCT03818061 (phase II)
6	Atezolizumab	Genentech (Roche)	IgG1	Programmed death-ligand 1 (PD-L1)	Humanized	NCT03708224 (phase II) NCT02423863 (phase II) NCT03818061 (phase II) NCT03829501 (phase I/II) NCT03452137 (phase III) NCT03289962 (phase I) NCT03212469 (phase I/II) NCT03170960 (phase I/II) NCT03313804 (phase II) NCT03841110 (phase I NCT03386721 (phase II) NCT03228667 (phase II)
7	Avelumab	Merck KGaA and Pfizer Inc	IgG1	PD-L1	Human	NCT03844763 (phase I/II) NCT03494322 (phase II) NCT04052204 (phase II) NCT03260023 (phase I/II) NCT02999087 (phase III) NCT03409458 (phase I/II) NCT03498378 (phase I) NCT02952586 (phase III/terminated due to lack of efficacy) NCT02554812 (phase II) NCT03228667 (phase II)
8	Tremelimumab	Pfizer and MedImmune (AstraZeneca)	IgG2	Cytotoxic T-lymphocyte-associated protein 4 (CTLA-4)	Human	NCT03019003 (phase I/II) NCT02551159 (phase III) NCT02999087 (phase III) NCT02369874 (phase III) NCT03283605 (phase I/II) NCT02319044 (phase II) NCT03212469 (phase I/II) NCT03426657 (phase II) NCT03624231 (phase II) NCT03518606 (phase I/II) NCT03292250 (phase II) NCT03522584 (phase I/II) NCT02643303 (phase I/II) NCT03509012 (phase I) NCT03529422 (phase I)
9	Varlilumab	Celldex Therapeutics and Bristol-Myers Squibb AB	IgG1	CD27 (TNFRSF7)	Human	NCT02543645 (phase I; terminated) NCT02335918 (phase I/II; completed)
10	Patritumab	Daiichi Sankyo Inc	IgG1	Receptor tyrosine-protein kinase erbB-3 (HER3)	Human	NCT02350712 (phase I; completed) NCT02633800 (phase II; terminated)
11	Durvalumab	MedImmune (AstraZeneca)	IgG1	PD-L1	Human	NCT03019003 (phase I/II) NCT03162224 (phase I/II) NCT02291055 (phase I/II) NCT02997332 (phase I) NCT02551159 (phase III) NCT03829007 (phase I/II) NCT02369874 (phase III) NCT03737968 (phase II) NCT03051906 (phase I/II) NCT03258554 (phase II/III) NCT03691714 (phase II) NCT02207530 (phase II) NCT02319044 (phase II) NCT03292250 (phase II) NCT02318277 (phase I/II) NCT02423863 (phase II) NCT03518606 (phase I/II) NCT02499328 (phase I/II) NCT04262388 (phase II) NCT03983954 (phase I) NCT03212469 (phase I/II) NCT03739931 (phase I)
12	Tomuzotuximab	Glycotope GmbH, Octapharma AG	IgG1	EGFR	Human	NCT02052960 (phase II)
13	Monalizumab	Innate Pharma SA and AstraZeneca	IgG4	CD94/NK group 2 member A (NKG2A)	Humanized	NCT02643550 (phase I/II)
14	Utomilumab	MorphoSys AG, Pfizer Inc	IgG2	CD137 (4-1BB)	Human	NCT02554812 (phase II)
15	Cixutumumab	ImClone Systems (Eli Lilly)	IgG1	Insulin-like growth factor 1 (IGF-1) receptor	Human	NCT00617734 (phase II; completed)
16	Duligotuzumab	Genentech (Roche)	IgG1	HER3	Humanized	NCT01911598 (phase I; completed)
17	PF04518600	Pfizer	IgG2	OX40 protein (CD134)	Human	NCT02315066 (phase I)
18	IPH2102 (Lirilumab)	Innate Pharma SA and Bristol-Myers Squibb AB	IgG4	KIR2DL1/2/3	Human	EU clinical trial: CA223-001 (phase I/II)
19	Spartalizumab	Novartis	IgG4	PD-1	Humanized	NCT04213404 (phase I) NCT04000529 (phase I)
20	Sym004 (two mAbs, futuximab, and modotuximab)	Symphogen A/S	IgG1	EGFR	Chimeric	NCT01417936 (phase II; completed)
21	Ficlatuzumab	AVEO Oncology	IgG1	Human hepatocyte growth factor/scatter factor (HGF/SF) ligand	Humanized	NCT03422536 (phase II)
22	ARGX110 (Cusatuzumab)	Argenx SE and Janssen Research & Development, LLC	IgG1	CD70	Humanized-defucosylated	NCT02759250 (phase I; completed)
23	Urelumab	Bristol-Myers Squibb AB	IgG4	CD137 (4-1BB ligand)	Human	NCT02110082 (phase I; completed)
24	Cemiplimab-rwlc	Regeneron and Sanofi	IgG4	PD-1	Human	NCT04242173 (phase II)
25	Dalantercept	Acceleron Pharma Inc	Fc of IgG1	Activin receptor-like kinase 1 (ALK1)	ALK1-Fc fusion protein	NCT01458392 (phase II; completed)
26	FRMD4A antibody	Cancer Research Technology	Not specified	FERM domain containing 4A (FRMD4A)	Not specified	Preclinical
27	Zalutumumab	Genmab A/S	IgG1	EGFR	Human	NCT00401401 (phase I/II; terminated) NCT00707655 (phase I/II; terminated) NCT01054625 (phase I/II; completed) NCT00542308 (phase II; completed) NCT00496652 (phase III; completed).
28	Nimotuzumab	CIMYM BioScience and Oncoscience AG	IgG1	EGFR	Humanized	NCT00957086 (phase III)
29	Daromun	Philogen SpA	ScFv	Extra-domain B (ED-B) of fibronectin (L19) and fibromun (L19-TNFalpha)	Fusion (A combination of darleukin (L19-IL2), fused to a human scFv	Preclinical
30	ABL001	ABL Bio	IgG1-ScFv	VEGF/DLL4 (Delta Like Canonical Notch Ligand 4)	Bispecific antibody (humanized bevacizumab and a Dll4-targeting ScFv)	NCT03292783 (phase I)
31	Panitumumab	Abgenix Inc and Amgen	IgG2	EGFR	Human	NCT02415881 (phase I) NCT03733210 (phase I) NCT03405142 (phase I)
32	Enoblituzumab	MacroGenics	IgG1	CD276 (B7-H3)	Humanized	NCT04129320 (phase II/III) NCT02475213 (phase I)
33	Bavituximab	Peregrine Pharmaceuticals	IgG1	Phosphatidylserine	Chimeric	NCT04150900 (phase I)
34	Telisotuzumab vedotin (ABBV-399)	AbbVie	IgG1	Tyrosine-protein kinase Met (c-Met)	Humanized ADC (Ab-MMAE)	Preclinical
35	Budigalimab (ABBV-181)	AbbVie	IgG1	PD-L1	Humanized	NCT04196283 (phase I) NCT03000257 (phase I)
36	Cosibelimab	Checkpoint Therapeutics	IgG1	PD-L1	Human	NCT03212404 (phase I)
37	CPI-006	Corvus Pharmaceuticals	IgG1	CD73 (NT5E: ecto-5′-nucleotidase)	Humanized	NCT03454451 (phase I)
38	Hu5F9-G4	Forty Seven, Inc.	IgG4	CD47	Humanized	NCT02953782 (phase I)
39	W0101	Pierre Fabre	IgG1	Insulin-like growth factor 1 receptor (IGF-1R)	Humanized ADC (Ab-auristatin)	NCT03316638 (phase I/II)
40	Indatuximab ravtansine (BT-062)	ImmunoGen	IgG4	CD138 (syndecan-1)	Chimeric ADC (Ab-ravtansine)	Preclinical
41	Tislelizumab (BGB-A317)	BeiGene	IgG4	PD-1	Humanized	NCT03430843 (phase III) NCT03783442 (phase III) NCT03957590 (phase III) NCT03924986 (phase III)
42	GBR 1372	Glenmark Pharmaceuticals	Not specified	EGFRxCD3	Bispecific antibody	Preclinical
43	ISU104	ISU ABXIS Co	Not specified	HER3	Human	NCT03552406 (phase I)
44	GA201 (RG7160):	Roche	IgG1	EGFR	Humanized	NCT00721266 (phase I; completed)
45	LJM716	Novartis AG	IgG1	HER3	Human	NCT01598077 (phase I; completed) NCT01822613 (phase I; completed)
46	Siltuximab	Centocor, Inc (Janssen Biotech).	IgG1	IL6	Chimeric	NCT00841191 (phase I/II)
47	Vopratelimab (JTX-2011)	Jounce Therapeutics, Inc.	IgG1	ICOS	Humanized	NCT04319224 (phase I/II) NCT02904226 (phase I/II)
48	Ipilimumab	Bristol-Myers Squibb	IgG1	CTLA-4	Human	NCT02812524 (phase I) NCT02919683 (phase II) NCT02741570 (phase III) NCT02823574 (phase II) NCT04080804 (phase II) NCT03690986 (phase I) NCT03700905 (phase III) NCT03162731 (phase I) NCT01935921 (phase I) NCT03003637 (phase I/II) NCT03406247 (phase II) NCT03620123 (phase II)
49	Trastuzumab	Genentech (Roche)	IgG1	HER2	Humanized	NCT00004163 (phase II) NCT02627274 (phase I)
50	Pertuzumab	Genentech (Roche)	IgG1	HER2	Humanized	NCT02465060 (phase II)
51	Onartuzumab	Genentech (Roche)	IgG1	c-Met	Humanized	Preclinical (Fab fragments with murine variable domains fused to human IgG1 constant domains)

Each listed antibody was described in term of type, class, targeted antigen, involved companies, as well as details of the clinical development stage based on information available on ClinicalTrials.gov.

Each listed antibody was described in term of type, class, targeted antigen, involved companies, as well as details of the clinical development stage based on information available on ClinicalTrials.gov.

### Anti-Epidermal Growth Factor Receptor Antibodies

EGFR is a glycoprotein belonging to the ErbB receptor family, and it is composed of an extracellular ligand-binding domain, an intracellular tyrosine kinase domain, and a hydrophobic transmembrane segment (36). Under unstimulated conditions, the EGFR is predominantly available as an auto-inhibited, dimerization-incompetent, state at the cell membrane (37). EGFR can bind to different ligands [transforming growth factor alpha (TGF-α), amphiregulin, and EGF] that can trigger receptor dimerization and subsequent auto activation of the tyrosine kinase from the intracellular domain of the receptor (38). These ligand-induced EGFR conformational changes can also recruit the endocytic machinery that facilitates receptor endocytosis, with ~10-fold higher internalization rates for ligand-bound than for unliganded EGFR (39).

EGFRs are expressed on the cell surface, and the mitogen-activated protein kinases (MAPK) pathway is the most important pathway in mediating the biological response of the EGFR (40). This pathway interacts with over a hundred substrates to propagate various physiological responses, such as growth, proliferation, differentiation, migration, and inhibition of apoptosis (41, 42). EGFR is normally expressed as 40,000 to 100,000 receptors per cell (43), whereas in 80–90% of HNC cases EGFR and TGF-α are overexpressed by 1.7-fold and 1.9-fold, respectively (44). EGF and EGFR exert a critical role in cellular growth and differentiation both in healthy and cancerous tissues (45). The anomalous activation of the EGFR generate improved proliferation and additional tumor-promoting activities in different types of cancer, including HNC (36). Overexpression of EGFR can happen at early stage carcinogenesis of the head and neck, and can rise progressively together with other histological abnormalities, from hyperplasia to dysplasia, *in situ* carcinoma, and invasive carcinoma (46). Therefore, EGFR was selected as a potential target for anti-cancer antibodies.

Therapeutic antibodies were developed to target the extracellular domain of EGFR as demonstrated by cetuximab. This strategy was designed to avert receptor activation by endogenous ligands via competitive inhibition. In addition, it can internalize the antibody-receptor complex, and successively downregulate the EGFR expression (36). As a mono-therapy, cetuximab generates a cytostatic rather than a cytotoxic effect, which affected the clinical trials outcomes (47). The EXTREME trial (48) concluded that cetuximab plus platinum–fluorouracil CT can enhance the overall survival by 2.7-month, and a 20% reduction in the relative risk of death, when compared to the chemotherapy-alone group. Secondary efficacy end points were also enhanced by the addition of cetuximab, demonstrated by a 2.3-month prolongation of progression-free survival. The best perceived response rate (20%) was at the lower end of the range usually reported for cisplatin-based therapy; this could be attributed to the fact that nearly one third of the patients received carboplatin, which is connected with lesser response rates than cisplatin (49, 50). The side effect profile in the chemotherapy alone group was typical of that for the combination of platinum plus fluorouracil (49, 51). These side effects are generally exemplified by neurotoxicity, ototoxicity, and renal toxicity (52). In 2006, cetuximab was approved by the FDA to treat HNCs, and currently being used for different indications (**Table 1**). The US patents of cetuximab expired in 2016 (EU patents expired in 2014) (53). North American marketing rights of biosimilar cetuximab were transferred from Bristol-Myers Squibb to Eli Lilly in 2015. In addition, different companies are developing biosimilar versions of cetuximab (**Table 2**).

**Table 2:** Cetuximab biosimilars.

	Product name	Development stage	Involved company
1	Cetuximab biosimilar ONS1055	Preclinical	Oncobiologics and Outlook Therapeutics
2	Cetuximab biosimilar RPH002	Phase III	R-Pharm
3	Cetuximab biosimilar ONS1055	Preclinical	Viropro, Oncobiologics, and Outlook Therapeutics
4	Cetuximab biosimilar ABP494	Preclinical	Actavis, Allergan, and Amgen
5	Cetuximab biosimilar HLX05	Preclinical	Shanghai Henlius Biotech Inc
6	Cetuximab biosimilar ABP494	Preclinical	Actavis, Allergan, and Amgen
7	Cetuximab platform	Research	PlantForm Corporation
8	Cetuximab biosimilar CT-P15	Research	Celltrion
9	Cetuximab biosimilar BNV003	Research	Bionovis SA
10	Cetuximab platform	Research	PharmaPraxis
11	Cetuximab biosimilar CMAB009	Phase I/II/III	Mabtech, Shanghai Zhangjiang Biotechnology, and Sinomab
12	Cetuximab biosimilar KL 140	Phase I/II/III	Sichuan Kelun Pharmaceutical Research Institute
13	Cetuximab biosimilar CDP-1	Phase I/II/III	Dragonboat Biopharmaceutical
14	Cetuximab biosimilar (STI-001)	Phase III	Mabtech
15	Cetuximab biosimilar	Research	BioXpress Therapeutics

**Figure 2:**
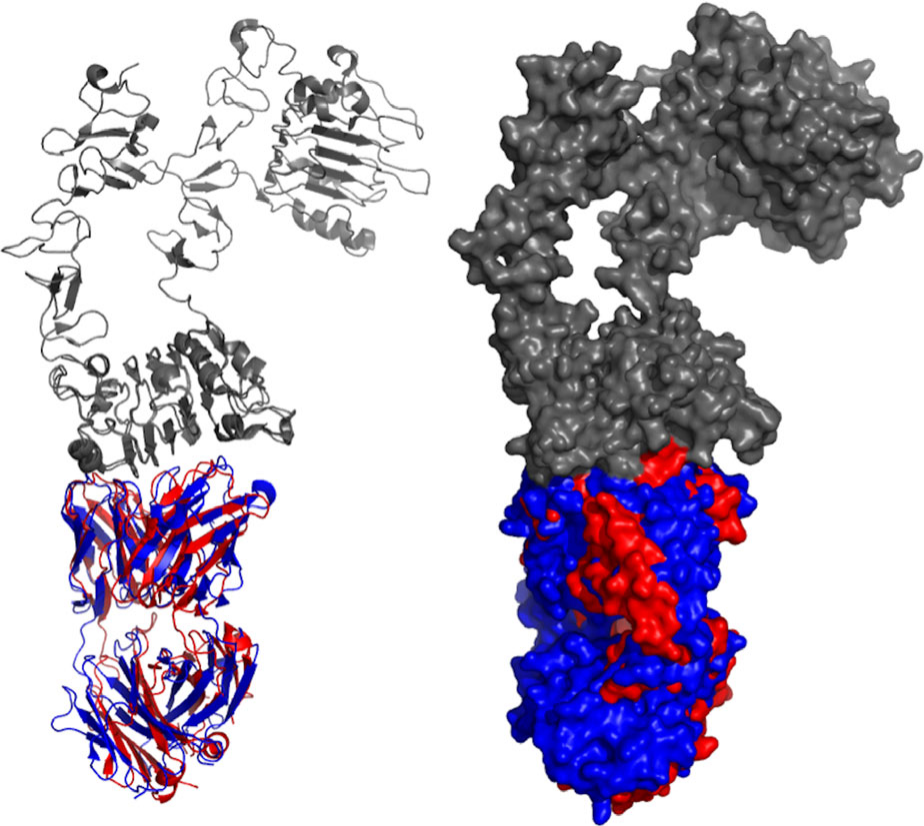
Aligned structures of panitumumab and cetuximab. Crystal structures of panitumumab (red) and cetuximab (blue) were aligned while binding to EGFR (gray). Both crystallized antibodies were in Fab format. Crystal structures were viewed and analyzed using PyMOL (The PyMOL Molecular Graphics System, Version 2.4.0 Schrödinger, LLC.)

Besides Cetuximab there are seven other antibody-based projects that are being developed against EGFR, including tomuzotuximab, Sym004, Zalutumumab, nimotuzumab, GA201 (RG7160), GBR 1372, and panitumumab (**Table 1**). The structures of nimotuzumab, panitumumab, and cetuximab were crystallized and deposited in the Protein Data Bank (PDB) as 3GKW, 5SX4, and 1YY9, respectively. Both panitumumab, and cetuximab were crystallized with EGFR, so their crystal structures were aligned to demonstrate the binding interaction (**Figure 2**). These two antibodies have shared and overlapped epitope on the EGFR surface.

### Immune Checkpoint Inhibitors

Circulating T lymphocytes normally examine the identity of other cells in the body to differentiate self from non-self antigens are therefore referred to as “checks.” This is achieved when a receptor binds to an equivalent ligand on a host cell, which will enable the T cells to identify it as a host cell, and prevents the triggering of an immune response (54). The main receptors involved in this type of immunological response are PD-1 (55), CTLA-4 (56), ICOS (57), OX40 (58), TIM-3 (59), and many others (60). We will focus on two main targets, PD-1/PD-L1 and CTLA-4, against which many anti-HNC antibodies are being developed.

PD-1 (CD279) is a type I transmembrane receptor with extracellular domains comparable with canonical immunoglobulin, and is responsible for signal transduction to intracellular domains (61). PD-1 is expressed at the cell surface of different cells, like T cells, monocytes, B cells, natural killer T cells, and dendritic cells (62). PD-1 receptor can interact with two ligands, PD-L1 (CD274) and PD-L2 (CD273), which are also expressed on cell surface like immunoglobulin transmembrane receptors (63). PD-L1 is controlled by external stimuli and constitutively present on both hematopoietic and non-hematopoietic cells (64), while PD-L2 is expressed inducibly on the surface of immune cells such as macrophages, dendritic cells, and mast cells (65). PD-1/PD-L1 system in cancer impedes proliferation of T lymphocytes, release of cytokines, and cytotoxicity, which empower cancer cells to deceive the host T cells, and evades an immune response that can potentially destroy these cells (66).

The initial immunotherapeutic agents to reveal indication of response durability and survival advantage in platinum-pre-treated recurrent and metastatic HNC are anti-PD-1 monoclonal antibodies (67, 68). In 2016, the FDA licensed nivolumab and pembrolizumab for the treatment of platinum-based therapy resistant patients (**Table 1**). CheckMate 141 and KEYNOTE-040 are two completed phase III randomized trials that have shown the high effectiveness of nivolumab and pembrolizumab, when compared to methotrexate, docetaxel, or cetuximab (13, 69, 70).

**Figure 3:**
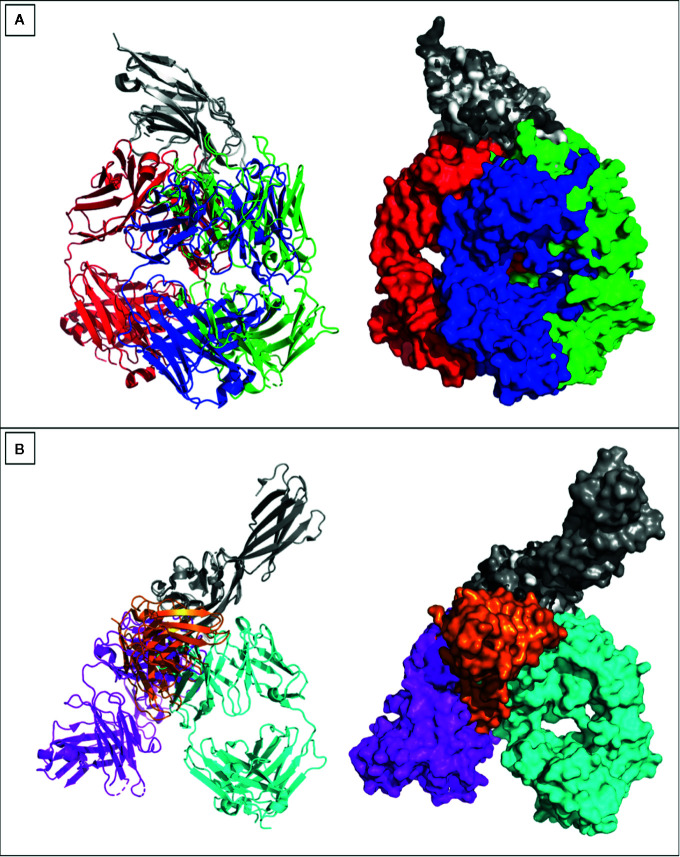
Crystal structures of anti PD-1 and PD-L1 antibodies. Crystal structures of **(A)** Pembrolizumab (red), toripalimab (green), nivolumab (blue) were aligned while binding to PD-1 (gray). **(B)** Atezolizumab (magenta), avelumab (orange), durvalumab (cyan) were aligned while binding to PD-L1 (gray). All crystallized antibodies were as Fabs apart from avelumab that was crystallized as single chain variable fragment (ScFv). Crystal structures were viewed and analyzed using PyMOL (The PyMOL Molecular Graphics System, Version 2.4.0 Schrödinger, LLC.)

The outcomes of the KEYNOTE-048 study have confirmed the substantial effect of anti-PD-1 in the first line recurrent and metastatic HNC setting (71). The enhancement in survival revealed in the KEYNOTE-048 study, and the toxicity of the EXTREME regimen, has encouraged the FDA to approve Pembrolizumab to be used with CT as a first-line treatment of all patients with recurrent and metastatic HNC. Pembrolizumab was approved in 2019 as a single agent in cases with a PD-L1 Combined Positive Score (CPS) ≥1 (72). In addition to these two approved antibodies, there are currently four antibodies underdevelopment against PD-1 receptor, including toripalimab, cemiplimab-rwlc, tislelizumab, and spartalizumab, while five other antibodies were directed against the ligand 1 (PD-L1), including atezolizumab, avelumab, durvalumab, budigalimab, and cosibelimab. Details of the aforementioned antibodies are summarized in **Table 1**. The PDB portal includes crystal structures of three anti-PD-1 antibodies pembrolizumab (PDB entry: 5GGS), toripalimab (PDB entry: 6JBT), and nivolumab (PDB entry: 5GGR). Similarly, three anti-PD-L1 antibodies were deposited in the PDB [atezolizumab (PDB entry: 5XXY), avelumab (PDB entry: 5GRJ), and durvalumab (PDB entry: 5X8M)]. The six antibodies have bound to distinct epitopes on each of the PD-1 and PD-L1, as can be noticed through divergence of their aligned structures (**Figure 3**).

The other checkpoint target is CTLA-4 (CD152). As a B7/CD28 family member, CTLA-4 can inhibit T cell functions (73). It is constitutively expressed by regulatory T cells (Tregs), and it can be upregulated upon activation by CD4^+^ T cells (74). CTLA-4 primarily compete with CD28 receptors for binding to B7 ligands (B7-2/CD86 and B7-1/CD80) on the antigen presenting cells (APCs) (75). B7 ligands on APCs binds to CD28 receptors on T-cells and provide the necessary second activation signal. Nevertheless, CTLA-4 receptors bind to B7 ligands with greater affinity and at a lesser surface density, and in that way surpass CD28 receptors for binding with B7 ligands. This inhibition of the second pathway would thus lead to anergy in T-cells (76).

**Figure 4:**
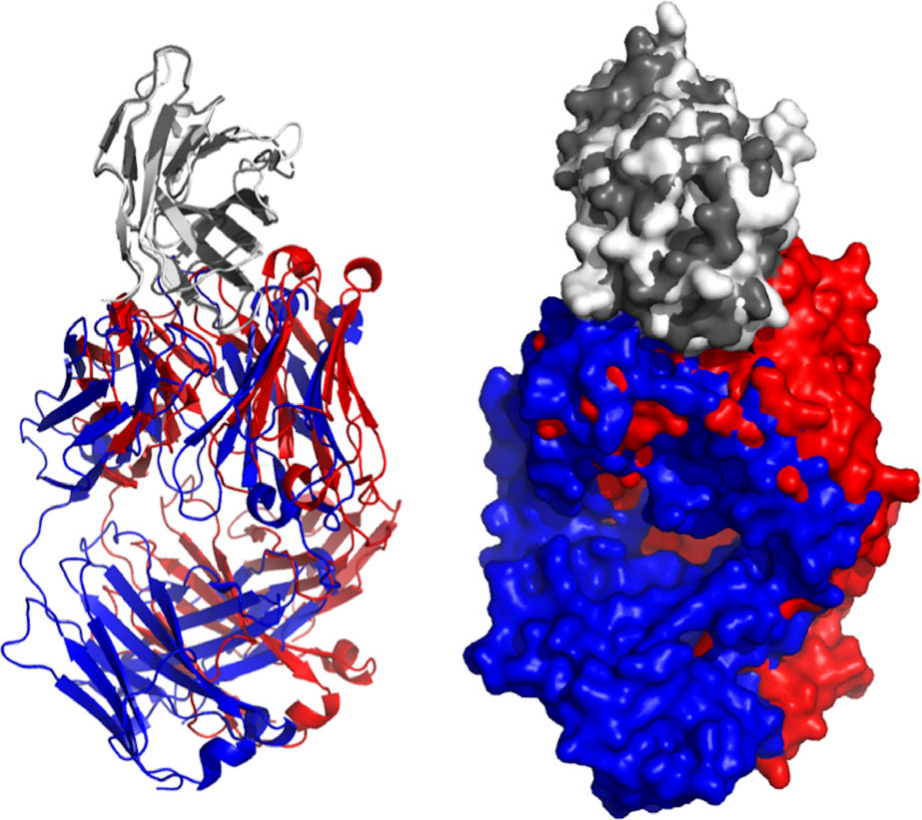
Aligned structures of Tremelimumab and Ipilimumab. Crystal structures of Ipilimumab (red) and tremelimumab (blue) were aligned while binding to EGFR (gray). Both crystallized antibodies were in Fab format. Crystal structures were viewed and analyzed using PyMOL (The PyMOL Molecular Graphics System, Version 2.4.0 Schrödinger, LLC.)

Human CTLA-4 includes a leader peptide and three domains: an extracellular V domain (116 amino acids), a transmembrane region (37 amino acids) and cytoplasmic tail (34 amino acid) that contains two tyrosine-based motifs (77). CTLA-4 has an essential function in controlling the immune responses in cancer and is contemplated as a prospective target against cancer. Preclinical studies have shown that the inhibition of CTLA-4 can enhance therapeutic immunity to cancer (78). At present, two antibodies (tremelimumab and ipilimumab) are being tested in clinical trials against HNC (**Table 1**). Crystal structures of these two antibodies (5GGV and 5TRU) were aligned to illustrate the binding model of shared epitope, but with slight deviation (**Figure 4**).

### Limitations of the Currently Offered Antibody-Based Approaches

Despite the great potential of biotherapeutic antibodies in treating various types of cancer, the scenario is slightly more complicated in HNCs. HNC is very costly to be treated, and in the USA for example, it is considered to be the most expensive cancer to treat, with assessed costs of $96,000–$150,000 for multimodality treatment (79). Antibodies against HNCs have been hindered with limitations related to efficacy and safety.

Regarding the anti-EGFR antibodies, despite the 80–90% overexpression of EGFR in HNC, Cetuximab therapy is potent in only 10–20% of HNC patients (80). Thus, the clinical responses to Cetuximab might not be completely related to the EGFR expression levels in HNC (81). This absence of correlation is analogous to what was noted in colorectal cancer patients treated with Cetuximab (82). Nevertheless, these observations should be carefully interpreted as they could be attributed to variations in the methods adapted to measure the expression level of EGFR, such as staining protocols, tissue fixation techniques, storage time of these tissues, and type of preservation reagents (82). Another factor could be related to the utilization of primary tumor tissue to establish the patient’s EGFR status, but it is the metastases stage, which is biologically distinct from the primary tumors, that is treated with cetuximab (83). Aside from these possible experimental variabilities, this lack of correlation could also lie in the prospective for cetuximab to induce antibody-dependent cell-mediated cytotoxicity (ADCC), which can lead to indirect antitumor activity by the recruitment of cytotoxic host effector cells such as monocytes and natural-killer cells (84). Consequently, the EGFR expression level does not currently seem to have a predictive value, and the future development of a consistent immunocytochemistry technique or a reverse transcription polymerase chain reaction are certainly required.

HNCs can additionally develop resistance to cetuximab (85). Patients that are primarily responsive to anti-EGFR therapy frequently show resistance during the treatment course (86). Various factors allied with resistance have been recognized, such as ubiquitination and trafficking (87), overexpression and amplification of ErbB2 (88), KRAS mutations (89), polymorphism (90), changes in the microenvironment (91), reformed expression levels of VEGF (92), and altered expression levels of STAT3 (93). This resistance process can be signaling based, leading to activation of HER2, HER3, insulin growth factor receptor (IGFR), and c-Met (94, 95).

Another anti-EGFR is panitumumab, which was tested on patients with recurrent or metastatic HNC in the SPECTRUM trial (phase III) that examined cisplatin and fluorouracil with/without panitumumab (96). Unlike the EXTREME study that tested cetuximab (48), the SPECTRUM trial on panitumumab has shown enhanced progression-free survival but not an overall survival. The noticed clinical variations between panitumumab and cetuximab exemplify the difficulty of obtaining a suitable anti-EGFR response in HNC. This complexity is not only limited to efficacy and resistance, but also extends to the appearance of various side effects.

EGFR is expressed at the epidermis basal layer, so it is anticipated that skin toxicity, such as acneiform eruptions, could be a major side effect (97). The dose-dependent rash was identified in over 50% of treated patients, especially on face, neck, retro-auricular area, shoulders, and the upper trunk (98). In addition, cetuximab, is associated with different adverse events when used as a second line single agent, including rash, acne, dry skin, nail disorder, fever, nausea, vomiting, dyspnea, and other infusion-related reactions (99). The higher incidences of severe radiation dermatitis were observed following the clinical introduction of concurrent RT and cetuximab (100). For example, the EXTREME trial has shown nine cases of sepsis (4%) in the cetuximab group, in contrast to one case (<1%) in the CT-alone group (48).

On the other hand, immune checkpoint inhibitors (ICIs) show promising clinical benefit when generally compared to anti-EGFR antibodies. However, they can also cause a distinctive spectrum of side effects, influencing almost any organ. Shah et al. (101), have conducted a systematic review analyzing databases on patients with HNC, treated with ICIs, who developed immune-related adverse events (irAEs). The authors have identified 46 treatment-related adverse events from the pooled 791 patients, with at least 12 having essential consequence to irAEs. The most noticed adverse effects in patients receiving PD-1 inhibitors were associated with the endocrine, cutaneous, and gastrointestinal systems.

Analysis of other clinical trials, which involved various ICIs, indicated that merely ~15% of patients with refractory/metastatic HNC attained curable remissions and extended survival (102). According to The National Institute for Health and Care Excellence (NICE) report on nivolumab (103), clinical trial evidence showed that nivolumab enhanced the general survival by 2.6 months when compared with docetaxel, cetuximab, or methotrexate. However, there is uncertainty regarding the longer-term survival advantage after 2 years. Also, there is an ambiguity about its advantage in tumors expressing <1% PD-L1 protein. Therefore, despite the current promising clinical outcomes from these ICIs, it is vital to note that some of the observed clinical responses happened in HNC patients who expressed negligible levels or no PD-L1 (13). Accordingly, the reason why not all patients show similar response to PD-1/PD-L1-targeted treatments are still undistinguishable. Furthermore, given the absence of any alternative treatment for HNC patients that are unresponsive to anti-PD-1 antibodies, there is a crucial unmet clinical requirement to find other choices if these resistance cases prevail.

## Future Perspectives Through Nanotechnology

Nanotechnology has rapidly progressed to provide pronounced promise in combating cancer (104). The large surface area to volume ratio of nanoparticles represents an important advantage (105). As this will enable the nanoparticle surfaces to be densely coated with antibodies, small molecules, peptides, or other molecules (106). Hence the coated moieties can bind and recognize specific cancer molecules through a multivalent effect that can enhance the efficacy and sensitivity of these conjugates. Therefore, nanotechnology-based diagnostics are being advanced as favorable methods (107).

Effective cancer treatments should distinguish between malignant from non-malignant cells, and to specifically destroy malignant cells (108). Nanoparticles can be used in this targeting process through passive or active approaches. The former is based on the enhanced permeability and retention effect of the cancer tissues that can lead to accumulation of these nanoparticles (109, 110). This high leakiness of tumor tissues is partially because malignant cells are not responsive to cell signaling required to organize vasculogenesis (111, 112). While active targeting involves specific recognition of antigens that are expressed on the surfaces of cancer cells to deliver these nanoparticles (113). In addition, surface modifications of nanoparticles can minimize uptake by the macrophage phagocytic system, and enhance circulation time (114). Consequently, nanoparticles can enhance the selectivity and effectiveness of these physical, chemical, and biological approaches to provoke cancer cell death, and with a minimum toxic effect on non-malignant cells. Different nanoparticles are currently in clinical trials for the treatment of and diagnosis of HNC (**Table 3**).

**Table 3:** Nanoparticles-based approaches in head and neck cancer.

	Nanoparticle (NP) type	Application	Responsible party	Development stage (ClinicalTrials.gov Identifier, active and recruiting)
1	Silica NP and experimental dye-labeled particle (dots), cRGDY-PEG-Cy5.5-C dots	Imaging	Memorial Sloan Kettering Cancer Center	NCT02106598 (phase I/II, recruiting)
2	Albumin stabilized NP and paclitaxel	Treatment	University of Southern California	NCT02495896 (phase I, active, not recruiting)
3	Albumin stabilized NP and paclitaxel	Treatment	Washington University School of Medicine	NCT01566435 (phase II, active, not recruiting)
4	Ferumoxytol (iron oxide) NP	Imaging	M.D. Anderson Cancer Center	NCT01895829 (phase I, active, not recruiting)
5	Silicon incorporated with quaternary ammonium polyethylenimine (PEI) NP	Device	Hadassah Medical Organization	NCT01007240 (phase I, unknown)
6	Hafnium oxide (HfO_2_) nanoparticle	Treatment	Nanobiotix	NCT01946867 (phase I, unknown)
7	Lipid NP encapsulating three mRNAs encoding human OX40L, interleukin 23 (IL23), and interleukin 36γ (IL36γ)	Treatment	Moderna Therapeutics	NCT03739931 (phase I, unknown)

Nanoparticles could be adapted as dendrimers, liposomes, polymers, iron oxide, nanotubes, nanowires, and gold nanoparticles (GNPs) (115). Colloidal GNPs exhibit a distinctive localized surface plasmon resonance (LSPR) when a precise light wavelength encounters electrons on the GNPs surface (116). The light generates a combined coherent oscillation of these electrons, leading to the successive extinction of light. Scattering and absorption of the light relies on the medium of the colloidal GNPs as well as on their physical dimensions (117). These novel physico-chemical properties of GNPs have generated significant attention for developing both diagnostic and therapeutic approaches against cancer (118, 119). The implementation of these GNPs in combating cancer could be demonstrated through their photothermal capability. Various review articles have comprehensively detailed the concept, applications, safety, heating and cells-death mechanisms, synthesis and morphology, *in vitro* and *in vivo* efficacy, and physicochemical properties of these therapies (120–127). However, the basic principles of this approach are summarized and illustrated in this section to provide the reader with brief outlines of this potential field.

The concept of photothermal therapy is based on the application of a laser light at a specific wavelength to the surface of GNPs, which can trigger the surface electrons to be excited and resonate strongly, and conversion of light into heat swiftly happens (128). This results in bubble creation that can kill cells more effectively. The implemented laser light could be used in both the visible and near-infrared (NIR) wavelength region, and as pulsed or continuous wave (CW) (122). For instance, CW laser can trigger elevation of the cells temperature in the range of 41–47°C for tens of minutes, and this is known as hyperthermia (129). This state can cause permanent destruction of the cells through proteins denaturation and/or cell membrane damage (130).

GNPs have been investigated in different sizes and morphologies, for example nanorods (GNRs), nanospheres (GNSs), nanostars, hollow nanoshells, nanorings, and nanocages (131–135). The LSPR peaks of GNPs can be additionally turned to NIR region (136). The light absorption efficiency of GNPs in this region is high (extinction coefficient: 10^−9^ M^−1^ cm^−1^), which enable the light to penetrate deeply into the tissues, and enhance the photothermal effect (137, 138). To modulate the LSPR to the NIR region, GNRs represent a great potential, due to their ability to present longitudinal and transverse surface plasmon absorption peaks, attributed to the length and diameter of the GNRs, respectively (139). This spectral location of the LSPR can be modified by altering the aspect ratio (AR) of GNRs (139). GNRs with different ARs generate different GNRs solutions’ colors because of alterations in their response to light in the visible light spectrum (140).

Both GNSs and GNRs were used to generate photothermal effect within the visible or NIR region, respectively. El-Sayed et al., have initially used anti-EGFR antibody conjugated to GNSs (141) and GNRs (142) to diagnose and treat oral tumor cells *in vitro* using CW laser (514nm argon laser for GNSs; 800nm Ti:sapphire laser for GNRs). They have shown that tumor cells targeted with the conjugates were destructed with 2–3 times lower laser power when compared to normal cells. In another study, a Ti:sapphire laser at 800 nm (100 femtosecond pulse duration, 1 kHz repetition rate) was implemented to photothermally destroy oral cancer cells treated with anti-EGFR antibodies conjugated to GNSs (143). Their results have demonstrated that the laser power required to destroy the cancer cells was nearly 20 times lower than that required to damage normal cells. Subsequently, various research articles have successfully tested the safety and efficacy of these GNRs based photothermal conjugates in mice, canines, and felines (144–146).

Despite the aforementioned advantages of GNPs, there is a possible long-term toxicity owing to sluggish tissue clearance, which is a factor that requires attention before GNPs can be used *in vivo* (147). Even with the assumption that these GNPs could accumulate passively at cancer cells due to extravasation of leaky tumor vasculature (148); it is still difficult to confirm this specificity and the overall elimination mechanism if GNRs were used alone. Low specificity could result in destruction of healthy tissues, which could produce additional side effects. Therefore, a more desired therapeutic outcome could be achieved through surface modification by coating and conjugation for precise targeting of these GNPs to reduce these side effects while maintaining their efficacy.

Another major limitation is related to cetyltrimethyl ammonium bromide (CTAB) that is broadly implemented in the synthesis of GNRs in seed-mediated growth (149, 150) or seedless technique (151). As a surfactant, CTAB binds to the GNRs surface and facilitates and stabilizes the formation of diverse structures (152). CTAB-stabilized GNPs can be cytotoxic, so they must be adjusted to evade cytotoxicity and inflammation. The cytotoxicity of CTAB could be avoided by replacing CTAB with other molecules like organothiol compounds (153), ligand exchange (154), or modifying the surface with polymer and silica-coating (155). In addition to the aforementioned surface modifications, GNPs can be coated with different materials to enhance the GNPs performance such as Bovine Serum Albumin (156), polyethylene glycol (157), and antibodies (158, 159). An alternative approach is also developed as GNRs free of CTAB (160, 161), which enhances the suitability of these GNRs for clinical settings.

The conjugation of GNRs to antibodies represents a great advantage, since it will combine the high specificity of the antibody with the enormous optical potential of GNPs. However, the antibody target that can be used in this targeting process is of crucial importance. In addition, these antibodies should be carefully conjugated to GNRs to avoid loss of targeting efficacy due to steric hindrance. The conjugation process should be directed to a specific region within the antibody using a suitable linker, without affecting the specific binding regions for target detection. Therefore the size, shape, and surface modification of the implemented GNRs are key factors that will complement the overall success of these therapies.

## Conclusions

HNC represents an immense clinical problem that requires special attention. The HNC treatment approaches are based on surgery, RT, CT, and biotherapeutic antibodies. Both RT and CT are well known for their severe side effects, and surgery can result in serious facial disfiguration and loose of ability to smell, speak, or taste. Antibodies have shown remarkable success in treating various types of cancer. Nevertheless, the complexity of HNC has relatively hindered this success. The three licensed antibodies against HNC are only being used in combination with other treatment modalities. Numerous monoclonal antibodies, ADCs, and bispecific antibodies are under development. The efficacy of these antibodies could be enhanced through conjugation to GNPs, and the generation of photothermal therapies. These photothermal therapies can specifically destroy cancer cells and treat HNC in an effective way. In order to gain a maximum photothermal effect the main three components (GNPs, linker, and the antibody) require careful optimizations.

## Author Contributions

The author confirms being the sole contributor of this work and has approved it for publication.

## Conflict of Interest

The author is employed by the company SiMologics Ltd.
